# Aggressive Surgical Treatment in Late-Diagnosed Esophageal Perforation: A Report of 11 Cases

**DOI:** 10.5402/2011/868356

**Published:** 2011-06-22

**Authors:** Rahim Mahmodlou, Isa Abdirad, Mohammad Ghasemi-rad

**Affiliations:** ^1^Department of General and Thoracic Surgery, Emam Hospital, Urmia University of Medical Sciences, Urmia 57/35, Iran; ^2^Department of Genetics, Motahhari Hospital, Urmia University of Medical Sciences, Urmia 57/35, Iran; ^3^Genius and Talented Student Organization, Student Research Committee (SRC), Urmia University of Medical Sciences, Urmia 57/35, Iran

## Abstract

*Introduction*. Esophageal perforation is a relatively uncommon and lethal disease usually resulting from endoscopic procedures. Delay in the diagnosis and treatment occurs in more than 50% of cases, leading to a mortality rate of 40% to 60%, but this rate decreases is 10%–25% if treatment is carried out within 24 hours of perforation. *Case Presentation*. To analyze the characteristics, etiology, site of perforation, presentation, time interval till diagnosis, treatment and outcome of patients with esophageal perforation. Over a five-year period, from October 2004 through March 2009, 11 patients with esophageal perforation were referred to the division of thoracic surgery of a tertiary referral hospital. In eight patients, perforations were thoracic with delayed diagnosis for at least 48 hours. Two patients had cervical esophageal perforation, and one patient had early-diagnosed Boerhaave's syndrome. Eight patients are alive after followup for a period ranging from eight months to five years. In the remaining three patients, cancer was the underlying disease and the reason of death. *Conclusion*. No patient with esophageal perforation should be deprived from surgical repair due to delayed diagnosis. All, except preterminal patients, should undergo exploration after resuscitation, and appropriate treatment should be carried out depending on the findings during operation. Aggressive treatment is necessary in the case of established mediastinitis.

## 1. Introduction

Esophageal perforation is a relatively uncommon disease with high rate of mortality and morbidity [1, 2]. The most common cause of this lethal disease is iatrogenic due to endoscopic procedures [3, 4]. It seems that the prevalence of esophageal perforation is increasing worldwide because of widespread use of endoscopy for diagnosis and treatment of esophageal diseases [5]. The importance of early diagnosis and prompt surgical treatment of perforated esophagus cannot be overstated [6]. Considering the rarity of this condition and its nonspecific presentations, delay in the diagnosis and treatment occurs in more than 50% of cases [7]. The mortality rate rises up from 40% to 60% if there is delay in diagnosis and initiation of optimal treatment, but this rate decreases to 10%–25% if treatment is carried out within 24 hours of perforation [7, 8]. Despite decades of clinical experiences and surgical innovations, the management of esophageal perforations remain challenging especially for late-diagnosed or missed esophageal ruptures, and there is no gold standard for the surgical treatment of choice [5, 9]. 

The aim of this study was to analyze the characteristics, treatment, and outcome of patients with esophageal perforation referred to our hospital during a 4-year duration.

## 2. Case Presentation

Between October 2004 and March 2009, 11 patients with esophageal perforations referred to our tertiary referral hospital were evaluated. Demographic data, etiology, primary false diagnosis, cause of perforation, presentation, type of operation, postoperative complication, hospital stay, and outcome for each patient were analyzed to evaluate the management.

In eight patients, perforations were thoracic with delayed diagnosis for at least 48 hours. Two patients had cervical esophageal perforation, and one patient was referred with Boerhaave's syndrome in the first 24 hours of diagnosis (Table 1). Seven patients were males and four were females with the age range of 10 to 88 years. The causes of perforation were foreign bodies in two (chicken and fish bones), endoscopic instrumentation in five, trauma in one, and spontaneous in three patients. The etiologies were instrumentation in four patients, cancer in three cases, and peptic stricture in one case. In the first patient, the primary diagnosis was acute myocardial infarction with three-day CCU stay after detection of hydropneumothorax in chest X-ray following development of respiratory distress. In three patients primary diagnosis was parapneumonic effusion and were treated with tube thoracostomy. In one of endoscopically perforated patients, the endoscopist suspected a tracheoesophageal fistula aside from the tumor in mid-esophagus and consulted with pulmonologist for fiber optic bronchoscopy. He was febrile at the time of examination by pulmonologist; chest X-ray was taken which showed right-sided hydropneumothorax. After insertion of chest tube and oral methylen blue administration diagnosis was confirmed in the 3rd day after endoscopy. In another patient the primary diagnosis was pancreatitis, and on the 4th day of admission, chest X-ray was taken due to suboptimal response to pancreatitis treatment and revealed pneumomediastinum. Review of history revealed that she had upper endoscopy a week before referring to emergency department. In the remaining two, diagnosis was delayed due to the distance between the village and the local hospital and from the local hospital to the referral hospital. In the three patients that were presented in the first 24 hours there was no primary false diagnosis. Diagnosis in two patients with cervical esophageal perforation was confirmed with contrast study in one patient and direct exploration in another. 

Two patients with cervical esophagus perforation were treated with primary repair and drainage and postoperative period was eventless. In one patient with thoracic perforation who presented in the first 24 hours, operation was primary repair and reinforcement with intercostal muscle flap without postoperative event.

In one of the patients with the diagnosis of complicated empyema, after tube thoracostomy in the local hospital, due to patient's disagreement, we only placed feeding jejunostomy, and the patient died after 2 weeks. In the second patient in this group who did not have obstructive pathology, and whose esophagus tissues seemed to be suitable for primary repair, we performed a primary repair and reinforcement with intercostal muscle flap, and he died with sepsis and leakage. 

In the remaining six patients we carried out trans-thoracic esophagectomy, mediastinal debridement when necessitated, and gastropharyngostomy in the neck. There was no hospital mortality in these cases (Table 2).

In two patients with late-diagnosed perforation, primary repair was carried out in one and jejunostomy in the other. Both died after two weeks. Two patients died after 18 and 24 months followup not due to surgical complication but due to underlying disease.

## 3. Discussion

The etiology of esophageal perforation in the majority of patients (76%) is either diagnostic or therapeutic instrumentation of the esophagus [1, 2]. Spontaneous perforation (Boerhaave's syndrome) accounts for 15%, foreign bodies 14%, and trauma 10% of cases [10]. Despite continuing advances in the intensive care support, the mortality ranges from 9% to 41% [5, 11]. There is little controversy over the importance of early diagnosis and initiation of optimal treatment of esophageal perforation [1, 9, 10, 12]. The route of diagnosis is also an issue in the management of esophageal perforation. However, due to relative rarity and its nonspecific presentations, delay in the diagnosis and treatment occurs in more than 50% of esophageal perforations [9]. Treatment of esophageal perforation, especially in late-diagnosed or missed ruptures still is a challenge, with controversy surrounding its optimal management [1, 5, 9].

The major problem is generally the reluctance of the surgeon to adopt an aggressive policy in the management of these injuries. At present, most surgeons recommend immediate surgical intervention once the patient's condition is stabilized, except under unusual circumstances when nonoperative strategy may be used [10].

Treatment options include nonoperative and operative procedures. Nonoperative management of esophageal perforation has been advocated in selected situations. Conservative therapy should not be used in patients who have free intrapleural perforation. Cameron proposed three criteria for nonoperative management: firstly perforation must be contained in the mediastinum and should be drained back into the esophagus, secondly there are mild symptoms, and thirdly there should be minimal evidence of clinical sepsis [3, 10, 13]. None of our patients matched with these criteria, and we did not manage any patient with this option. 

Operative strategies can be further subdivided into primary repair with or without reinforcement, simple drainage of thoracic cavity, exclusive diversion operation, occlusion of perforation site with a prosthesis, and esophageal resection with or without reconstruction [9, 10, 14].

The choice of operative strategy depends on the cause, location of injury, underlying esophageal diseases, and time interval after perforation, extension of spillage, edge of wound, the age, and presence of any comorbidity [1, 14]. 

Although many believe that primary repair is gold standard for patients who present within the first 24 hours following perforation [13], primary repair can be done regardless of time interval between perforation and treatment if esophageal tissue is repairable and wound edges are viable after necrosectomy, there is no distal obstruction, and the size of defect is not greater than one-third of the circumference of the esophagus [14, 15]. Importantly, the greater the delay in the diagnosis of perforation, the more edematous and necrotic is the esophageal wall. In this circumstance, identification of the esophageal wall can be extremely challenging during dissection and attempt at direct repair frequently fails [14, 16]. We used this method in four patients, two with cervical and two with thoracic perforations. 

One of these was diagnosed within 24 hours and the other after 48 hours. The latter case which was our first patient among late-diagnosed thoracic esophageal perforations, treated with primary repair and reinforcement with intercostals-muscle flap was complicated with leakage and died from multiorgan failure and ongoing sepsis.

Even in more specialized centers, leaks at the site of primary repair have occurred in 20–30% of cases. Hospital mortality and delayed esophagectomy rate range from 14% to 11%–20%, respectively [12]. We believe that primary repair has no place in the treatment of late-diagnosed thoracic esophageal perforation in the presence of established mediastinitis, edematous esophagus, and fibrin deposition. 

The major shortcoming of conservative treatment of the esophageal perforation including drainage, endoprosthesis insertion and various forms of esophageal diversion or exclusion in late-diagnosed thoracic esophageal perforations and established mediastinitis is leaving necrotic esophagus and infected periesophageal tissues, and continuous soilage is a perplexing problem [10]. In addition, an often-difficult reconstructive surgery is inevitable [1, 14]. 

An alternative to exclusion and diversion is T-tube drainage of the perforation, creating a controlled esophagocutaneous fistula. T-tube placement can be used in high-risk patients, but continuous leakage can progress to sepsis and is often not recommended as routine procedure [15]. None of patients was treated with either of these two options.

Although esophageal resection is a major intervention, it is safe and reliable treatment option for complicated perforation of the thoracic esophagus. During the procedure, intrathoracic sepsis can be eliminated confidently, as its source, the leaking esophagus, is removed [14, 16]. Primary esophageal resection is indicated in the following circumstances: concurrent obstructive esophageal disease is detected, the injury is relatively extensive and associated with mediastinal or intrapleural sepsis, the viability of the wound edges, principally the mucosa, is in doubt, primary over swing of the perforation would result in at least 50% narrowing of the esophageal lumen, and generalized sepsis has already developed [1, 5, 9, 14, 16]. After resection of necrotic esophagus, an important question is carrying out immediate or delayed reconstruction. Delayed reconstruction advocates claim that delaying the reconstruction reduces the time it takes to complete the first surgical procedure and facilitates the resolution of the septic state [5]. We believe that resection of the thoracic esophagus with delayed reconstruction necessitates the exploration of the neck for cervical esophagostomy, laparotomy for decompressive gastrostomy, feeding jejunostomy, and ligature of the cardia. In reconstructive operation, instead of gastrostomy and ligature of the cardia, we did gastrolysis and an anastamosis in the clean cervical region. From time point of view, maximum difference between ligature of cardia, gastrostomy and creation of cervical esophagostomy and gastrolysis and cervical anastamosis in six consecutive cases was about 30 minutes. 

(Instead of stomach act as vascularized flap in mediastinum, second operation is very difficult due to adhesion in abdomen and conduit must be transpositioned in non physiologic route: substernal or transthoracic.)

We have done this aggressive option in eight cases of late-diagnosed (>48 hours) thoracic esophageal perforation without mortality.

It is our routine approach to the esophageal perforation that except in preterminal patients, we explore every thoracic esophageal perforation regardless of elapsed time between perforation and diagnosis and if criteria are not suitable for primary repair we do esophagectomy and debridement of necrotic tissues and decortication of lung if needed and in the same time reconstruct gastrointestinal tract with cervical gastropharyngostomy.

## Figures and Tables

**Table 1:** Demographic data, primary diagnosis, delay in diagnosis, route of diagnosis, and site of perforation, sepsis, and etiology in 11 cases of esophageal perforation.

Case no.	Sex	Age (years)	Primary Dx	Delay in Dx (hour)	Route of definite Dx	Site of perforation	Sepsis	Etiology
1	Male	60	Acute myocardial infarction	72	Contrast study	Middle third	Yes	Chicken bone
2	Male	48	Complicated empyema	8 days	Contrast study	Middle third	Yes	Spontaneous
3	Female	71	Pain after dilatation	48	Contrast study	Lower third	Yes	Instrumentation SCC**
4	Female	73		48	Contrast study	Middle third	Yes	Instrumentation (peptic stricture)
5	Female	66	T.E.F^±^	72	Contrast study	Middle third	Yes	Instrumentation SCC^†^
6	Male	10	—	24	Contrast	Cervical	No	Fish bone
7	Female	60	Pancreatitis	4 days	Contrast	Lower third	Yes	Instrumentation adenocarcinoma
8	Male	88	Empyema	6 day	Oral Methylene Bleu	Middle third	Yes	Instrumentation SCC^†^
9	Male	80	Empyema	10 days	Methylene Bleu	Middle third	No	Spontaneous
10	Male	52	—	12	Contrast	Lower third	Yes	Spontaneous
11	Male	28	—	12	Direct exploration	Cervical	Yes	Penetrating trauma

SCC: squamous cell carcinoma, Dx: diagnosis, TEF: transesophageal fistula.

**Table 2:** Type of operation, complication, hospital stay and patients outcome in 11 cases of esophageal perforation.

Case no.	Type of operation	Complication	Hospital stay (day) ICU	Outcome at time of reporting
1	T.T.E + reconstruction with stomach^±^	Leakage of anastamosis − −	8/25	Alive (5 years)
2	Primary repair with reinforcement	Sepsis and death		
3	T.T.E + reconstruction with stomach	None	5/13	Died after 2 years
4	T.T.E + reconstruction with stomach	None	6/14	Alive (3 years)
5	T.T.E + reconstruction with stomach	None	6/15	Died after 18 months
6	Primary	None	2/7	Alive (3 years)
7	T.T.E + reconstruction with stomach	Delayed extubation (tracheostomy)	30/43	Alive (8 months)
8	Chest tube and jejunostomy tube			Expired two weeks after
9	T.T.E + reconstruction with stomach	ARF^†^—delayed extubation	21/36	Alive (12 months)
10	Primary repair + reinforcement with intercostal muscle flap	None	3/10	Alive (14 months)
11	Primary repair and drainage	None	2/7	Alive (10 months)

^†^Acute renal failure, ^±^transthoracic esophagectomy and reconstruction with stomach.
